# Effect of Polyoxymethylene Fiber on the Mechanical Properties and Abrasion Resistance of Ultra-High-Performance Concrete

**DOI:** 10.3390/ma16217014

**Published:** 2023-11-02

**Authors:** Lixin Tan, Jun Yang, Chuanxi Li, Gaozhan Zhang, Qingjun Ding, Daosheng Sun, Yongyuan Zhang

**Affiliations:** 1 School of Civil Engineering, Changsha University of Science & Technology, No. 960, SEC. 2, Wanjiali South Road, Yuhua District, Changsha 410114, China; 2 Poly Changda Co., Ltd., No. 942, Guangzhou Avenue, Tianhe District, Guangzhou 510640, China; 3 Advanced Building Materials Key Laboratory of Anhui Province, Anhui Jianzhu University, No. 292, Ziyun Road, Shushan District, Hefei 230601, China; 4 School of Materials Science and Engineering, Wuhan University of Technology, No. 122, Luoshi Road, Hongshan District, Wuhan 430070, China

**Keywords:** ultra-high-performance concrete, polyoxymethylene fiber, mechanical strength, impact and abrasion resistance, flexural toughness

## Abstract

It is necessary to prepare marine UHPC with synthetic fibers instead of steel fibers, owing to the corrosion risk of steel fibers in marine environments. Currently, the performance of UHPC prepared with different types of fibers has not been comparatively investigated. This work prepared UHPC with steel fiber, polyoxymethylene (POM) fiber, polypropylene (PP) fiber, and polyvinyl alcohol (PVA) fiber. The effects of different fibers on the mechanical properties, impact, and abrasion resistance of UHPC were studied and compared. The results showed that increasing POM fiber can increase the mechanical strength, flexural toughness, impact, and abrasion resistance of UHPC. When its content reaches 2%, the adsorbed-in-fracture energy and abrasion strength of UHPC are 2670 J and 105 h/(kg/m^2^), respectively. At the same fiber content, POM fiber-reinforced UHPC shows better mechanical strength, toughness, and impact- and abrasion-resistance than the polypropylene (PP)- and polyvinyl alcohol (PVA)-fiber-reinforced UHPCs. Microstructure investigation found that PP fiber has the weakest binding with UHPC paste, which would directly pull out of the matrix under external tensile loading. This weak connection limits the strengthening and toughening effect on the UHPC. PVA fiber has an excellent interfacial connection with the UHPC paste. However, the low tensile strength of PVA fiber limits the strength and toughness of UHPC. POM fiber has a high tensile strength and can absorb tensile loading through debonding, fracture, and tearing. The fracture interface of POM fiber is large, indicating its significant role in strengthening and toughening the UHPC.

## 1. Introduction

With the advancement of economic development and marine development strategies in countries around the world, the construction of significant civil engineering projects, such as cross-sea bridges connecting islands and coasts, offshore terminals, offshore wind power bases, and artificial islands, is steadily being planned and carried out. As the most highly used material by mankind, concrete production consumes a high amount of mineral resources and energy. Many scientists have investigated the application of industrial waste in concrete preparation to lower its carbon footprint, for example, the extensive incorporation of fly ash, slag powder, and silica fume in concrete. Additionally, attempts at other solid waste replacements for cement have also been made. Gautam et al. [1,2] studied the fluidity, mechanical strength, and durability of concrete with bone china waste powder (BCWP) and granite cutting debris (GCD) replacement. They reported that concrete with 10% of BCWP and 30% of GCD replacement for cement and fine aggregate shows the highest strength, which can reduce the cost and carbon footprint for concrete production.

On the other hand, the design of a highly durable concrete material can also achieve the sustainability of the construction field. Concrete in the harsh marine environments of high salt, high humidity, salt fog, and tides is vulnerable to deterioration [3,4,5]. Under salt fog, waves, and freeze–thaw cycles, concrete materials can deteriorate, leading to structural damage to marine infrastructure [6]. Meanwhile, the scouring effect of sand-containing surges on concrete buildings in offshore areas leads to the abrasion and reduction in the protective layer for steel bars [7], which accelerates the exposure of the steel reinforcement. Under the corrosion of the ocean environment, ordinary steel rusts within one year, with a corrosion rate of 100 μm/year [8]. China’s Ministry of Transportation investigated many seaports and wharves throughout the country and found that a steel-reinforced concrete structure has an average service life of only 25 years. The direct annual economic loss caused by concrete deterioration is nearly 500 billion yuan [9]. Therefore, developing highly durable and abrasion-resistant concrete materials for marine engineering is related to its economy, safety, and durability. This measure is also an effective technical solution for the sustainable development of the modern marine industry.

Impact and abrasion resistance are crucial properties of marine concrete, as they determine the durability of marine concrete, the safety of the overall structure, and the cost of periodic maintenance. Ultra-high-performance concrete (UHPC) has the features of high compactness, ultra-high strength, and high toughness and is an excellent abrasion- and shock-resistant material for construction [10]. Therefore, using UHPC as a structural material for buildings subjected to seawater scouring offshore has a promising future. Generally, UHPC includes a high number of steel fibers, which can significantly enhance the toughness and ductility of UHPC [11]. However, steel fibers in UHPC may be at risk of corrosion in marine service environments. Shin et al. [12] found that chloride ions could corrode steel fibers through microcracks, decreasing the energy absorption capacity of UHPC under tensile loading. Longer immersion durations generally lead to a higher corrosion degree of fibers. Algaifi et al. [13] and Hearn et al. [14] have also reported that microcracks facilitate the invasion of harmful ions into the concrete and cause the erosion of steel fibers. Moreover, randomly distributed steel fibers may come into contact with steel bars in the UHPC structure, which would generate electrochemical reactions and then accelerate the erosion of UHPC. These reactions could lead to even more severe safety hazards [15,16].

Scientists used to prepare marine UHPC with synthetic fibers instead of steel fibers to address the risk of corrosion of steel fibers. For example, Dapper et al. [17] prepared UHPC with steel and polypropylene (PP) hybrid fibers and studied its ballistic impact resistance. The results showed that the fiber ratio did not significantly affect spalling and penetration depth for UHPC specimens. Deng et al. [18] investigated the stress–strain behavior of hybrid fiber UHPC. They concluded that PP fiber with a high aspect ratio can effectively restrain the growth of micro-cracks, thus improving the toughness of the UHPC sample. Li and Deng [19] compared the tensile failure behavior of UHPC strengthened with steel or hybrid fibers. They found that hybrid-fiber-reinforced UHPC shows better toughness damage characteristics than the single-steel-fiber-reinforced UHPC. Among those hybrid fibers, steel-polyvinyl alcohol (PVA) fiber presents higher efficiency in reinforcing UHPC than steel-polypropylene (PP) and steel-polyester (PET) fiber. Mosavinejad et al. [20] researched the effect of the incorporation of short PVA fibers on the durability of UHPC. They found that the addition of PVA fibers can restrain chloride diffusion and penetration in the UHPC. Recently, a new polyoxymethylene (POM) fiber with superior mechanical strength and corrosion resistance [21] has been developed. Researchers have utilized this fiber to produce concrete materials. Xue et al. [22] investigated the mechanical properties of reinforced seawater sea–sand concrete (SWSSC). They found that POM-fiber-reinforced concrete has a better early-age cracking resistance and mechanical strength than plain concrete. Wang et al. [23] studied the effect of POM fiber on the fatigue properties of airport pavement concrete. They concluded that POM fiber can increase the deformation capacity of the concrete but considerably shorten the fatigue life of concrete.

This paper aims to prepare UHPC with POM fiber and study the effect of POM fiber on the mechanical properties, impact, and abrasion resistance of UHPC. The results are compared with other kinds of fibers to reveal the toughening mechanism of POM fiber. The results can provide a theoretical basis for preparing corrosion- and abrasion-resistant UHPC.

## 2. Raw Material and Experiment

### 2.1. Raw Material

To enhance the abrasion resistance of the UHPC, this experiment used an abrasion-resistant high-ferrite cement [24,25] to prepare UHPC. The cement was produced by Guangxi Yufeng Cement Plant. The specific surface of the silica fume used in the experiment is 20,300 m^2^/kg according to Chinese Standard GB/T 19587-2017 [26], and the SiO_2_ content is 89% according to Chinese Standard GB/T 18736-2002 [27]. The fly ash used was fly ash microbeads produced by Sichuan Langtian Resources Comprehensive Utilization Co., Ltd. (Chengdu, China). It has a 28 d activity index of 109% and a water requirement ratio of 101% according to Chinese Standard GB/T 1596-2017 [28]. The apparent density of cement, silica fume, and fly ash are 3100 kg/m^3^, 2390 kg/m^3^, and 2510 kg/m^3^, respectively. The chemical composition of the cementitious materials is given in Table 1. The fine aggregate was quartz sand with an apparent density of 2650 kg/m^3^ according to Chinese Standard GB/T 14684-2011 [29]. It includes grains of particle sizes of 20–40 mesh, 40–80 mesh, 80–120 mesh at the weight proportion of 3:5:2, respectively. The PCA-I type of polycarboxylic acid superplasticizer produced by Sobute New Materials Co., Ltd. (Nanjing, China) was used in this experiment, which has a solid content of 45% and a water reduction rate of 35%.

**Figure 1:**
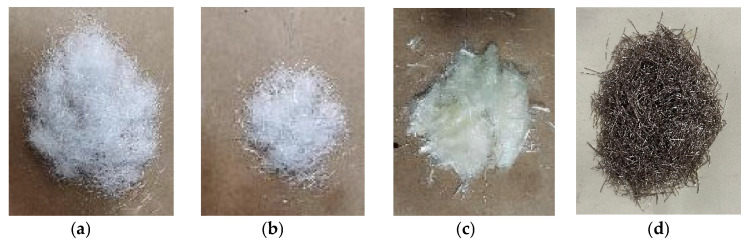
Morphology of fibers. (**a**) POM Fiber; (**b**) PP Fiber; (**c**) PVA Fiber; (**d**) Steel Fiber.

This experiment selected four kinds of fibers as the toughening phase of UHPC, including three synthetic fibers (PVA fiber, PP fiber, and POM fiber) and steel fiber. The morphology and parameters of various fibers are shown in Figure 1 and Table 2.

### 2.2. Sample Preparation

To investigate the effect of POM fibers on the properties of UHPC, this work first investigated the effect of fiber content and then the effect of fiber type on the properties of UHPC. The mixing ratios of UHPC samples are shown in Table 3. The sample preparation process was as follows: Firstly, the raw materials were weighed and put into the mixer to pre-mix for 1~2 min. Superplasticizer and 70% water were added, and the mixture was stirred for 3~5 min. Subsequently, fiber and the rest of the water were slowly added, and the mixture was continuously stirred for 3 min. Finally, the mixture was cast into a mold and covered with an impermeable film. After 1 d of sealed curing, the hardened samples were put into the standard curing room. The samples were cured until the specified age for performance testing.

### 2.3. Testing Method

#### 2.3.1. Workability and Mechanical Properties

The workability of UHPC was tested according to the requirements in Chinese Standard GB/T 50080-2016 [30]. The compressive test was conducted according to Chinese Standard GB/T 50081-2019 [31]. The 28 d compressive strength test was performed on a batch of three 100 mm × 100 mm × 100 mm UHPC samples and the results were averaged. The loading rate was set to 1.2 Mpa/s.

#### 2.3.2. Flexural Toughness

**Figure 2:**
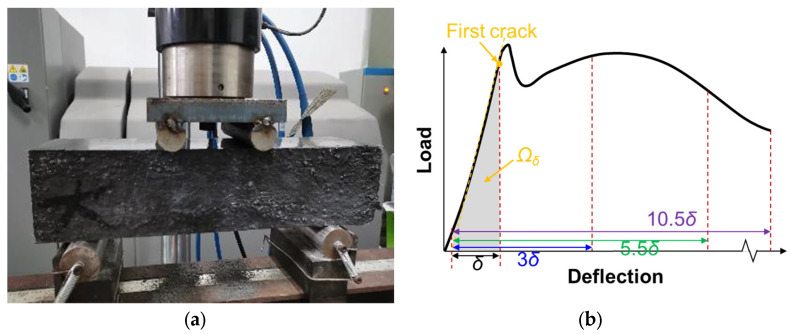
(**a**) Four-point flexural toughness test. (**b**) Calculation method of toughness indices.

According to Chinese Standard CECS13-2009 [32], four-point flexural toughness tests was taken on the UHPC using the MTS-300 kN (Eden Prairie, MN, USA), as depicted in Figure 2a. The bending tests were performed on 100 × 100 × 400 mm prismatic specimens of 28 d curing age, with a loading rate of 0.1 mm/s. The distance between the supporting rollers was 300 mm and the two loading rollers were located at equidistant points on the span. The load–deflection curves were obtained using a computer. The toughness indices *I*_5_, *I*_10_, *I*_20_ of UHPC were calculated using the method defined in Chinese Standard CECS13-2009 [32]. According to the Standard, the toughness indices are defined by Equations (1)–(3):

(1) $$I_{5}=\frac{\Omega_{3\delta }}{\Omega_{\delta }}$$

(2) $$I_{10}=\frac{\Omega_{5.5\delta }}{\Omega_{\delta }}$$

(3) $$I_{20}=\frac{\Omega_{10.5\delta }}{\Omega_{\delta }}$$

where *δ* is the deflection value at first-peak load, and 3*δ*, 5.5*δ,* and 10.5*δ* are deflection values 3, 5.5, and 10.5 times that of first-peak deflection, respectively. *Ω*_δ_ denotes the integral area of load–deflection curve from 0 to δ deflection. *Ω*_5.5*δ*_ and *Ω*_10.5*δ*_ denote the integral areas of ranges of 0–5.5*δ* and 0–10.5*δ*, respectively. These variables are visually characterized in Figure 2b. The residual strength factors *R*_5,10_ and *R*_10,20_ of UHPC are the values of 20 × (*I*_10_ − *I*_5_) and 10 × (*I*_20_ − *I*_10_), respectively. For every mixing proportion, three tests were conducted to obtain an average result.

#### 2.3.3. Abrasion Resistance Test

**Figure 3:**
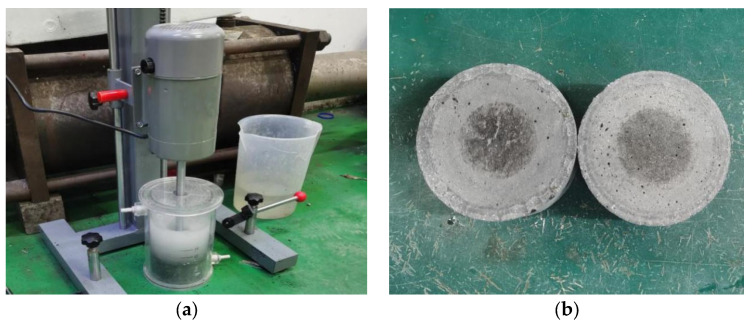
(**a**) Underwater steel ball method. (**b**) Samples after abrasion test.

The abrasion resistance was tested using the underwater steel ball method defined in Chinese Standard DL/T 5150-2017 [33]. The test equipment is shown in Figure 3a. The abrasion time was 72 h at a rotation speed of 2400 r/min. The sample size was Φ100 mm × 50 mm, with its appearance after the test given in Figure 3b. Three tests per series were conducted at 28 d age. The abrasion resistance of UHPC was calculated using Equation (4).

(4) $$R_{a}=\frac{tA}{\Delta M}$$

where *A* is the abrasion area of the specimen, Δ*M* is the mass difference before and after the test, and *t* is the testing time.

#### 2.3.4. Impact Resistance Test

Drop-weight impact resistance test was implemented according to American Concrete Institute (ACI) 544 [34]. The mass of the drop hammer in the test was 2.5 kg, and the drop height was 0.4 m. The size of the specimen was Φ100 mm × 50 mm. Every batch containing three samples was tested to obtain an average result. Absorbed-in-fracture energy for UHPC samples was calculated using Equation (5):

(5) W=Nmh

where *N* is the number of the impact test until sample fracture, *m* is the mass of the drop hammer, and *h* is the drop height.

#### 2.3.5. SEM-EDS

Quanta FEG450 scanning electron microscope (SEM) from FEI Company (Hillsboro, OR, US) was used to observe the interfacial bonding between fibers and the UHPC matrix to study the toughening mechanism of fibers on UHPC materials.

## 3. Results and Discussion

### 3.1. Effect of POM Fiber Content

#### 3.1.1. Effect of POM Fiber Content on Workability

**Figure 4:**
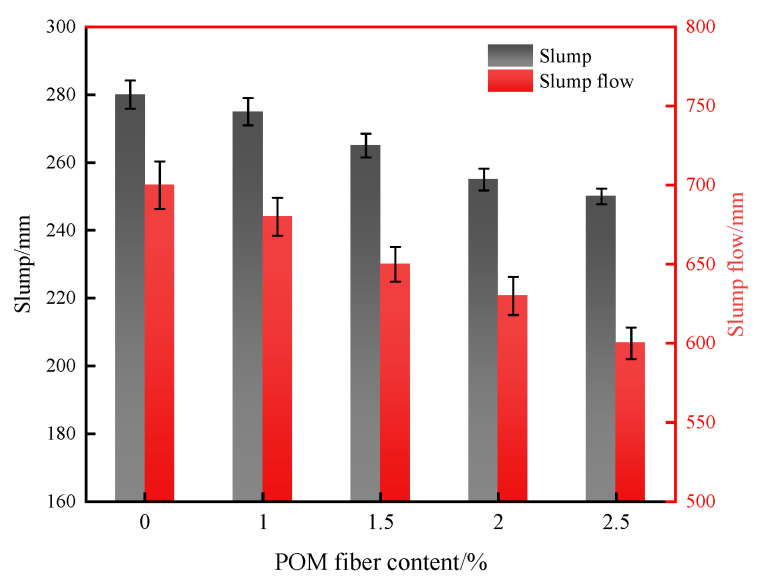
Effect of POM fiber content on the flowability of UHPC.

Since UHPC contains a high number of cementitious materials and a low water-to-binder ratio, all the fresh UHPC with different amounts of POM fiber in this work has good cohesion and water retention capacity. Figure 4 shows the slump and slump extension of UHPC with different contents of POM fiber incorporation. The optimal workability of UHPC without POM fiber addition was 280 mm of slump and 700 mm of slump extension. With the increase in POM fiber from 0% to 2.5%, the slump of UHPC decreased by 10.7% and the slump extension decreased by 14.3%. The flowability of UHPC decreased with increasing volume of the POM fiber. The main reason for this phenomenon is that the increasing fibers enlarge the friction inside the paste, increasing the energy barrier for the fresh flow of UHPC [35]. Furthermore, an increased amount of fiber would strengthen the bridging phenomenon between the fibers, forming a 3D network structure and hindering the flow of the UHPC paste.

#### 3.1.2. Effect of POM Fiber Content on Mechanical Strength

**Figure 5:**
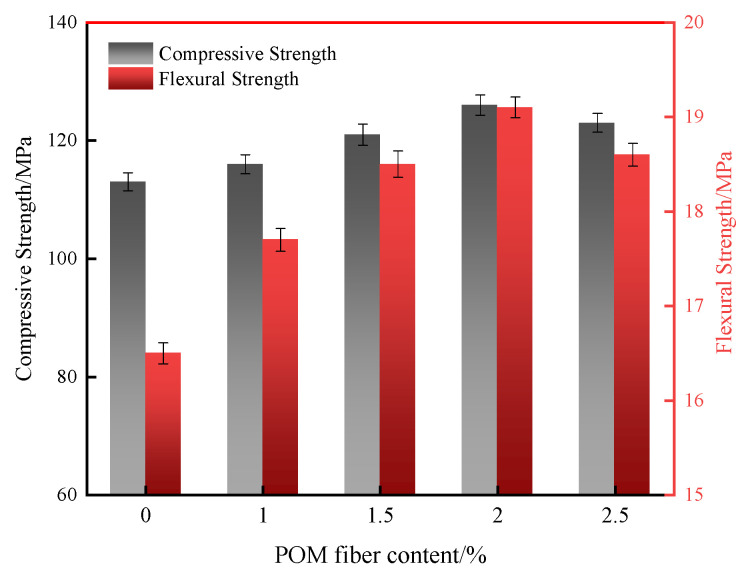
Effect of POM fiber content on the mechanical strength of UHPC.

The effect of POM fiber amount on the 28 d compressive and flexural strength of UHPC is shown in Figure 5. With the increase in POM fiber amount, both the compressive strength and flexural strength of UHPC show the tendency of an increment followed by a decrement. The compressive and flexural strengths of UHPC reach the maximum at a POM fiber content of 2.0%, with values of 126 MPa and 19.1 MPa, respectively. The reason is that the POM fibers have a high elastic modulus and tensile strength and disperse uniformly in the UHPC, which can restrain lateral deformations when the specimen is under compressive loading. The bridging effect of the fiber can inhibit the generation and growth of cracks in the UHPC and thus increase its flexural strength. However, when the fiber content is too high, the fibers would agglomerate and introduce defects such as air voids and microcracks in UHPC [36]. This would weaken the interfacial bond between the fiber and matrix, which offsets the reinforcing effect of the fibers and causes decreasing mechanical properties of UHPC. It should be noted that the enhancement in the flexural properties of UHPC through the addition of POM fibers is more significant than that of the compressive strength. This is mainly because of the smaller elastic modulus of POM fibers than the cement matrix, which leads to the enhancement in compressive properties not being noticeable. However, the network structure formed by POM fibers in the UHPC matrix can improve its flexural properties through bridging and the energy absorption of pulling out and breaking.

#### 3.1.3. Effect of POM Fiber Content on Impact and Abrasion Resistance

**Figure 6:**
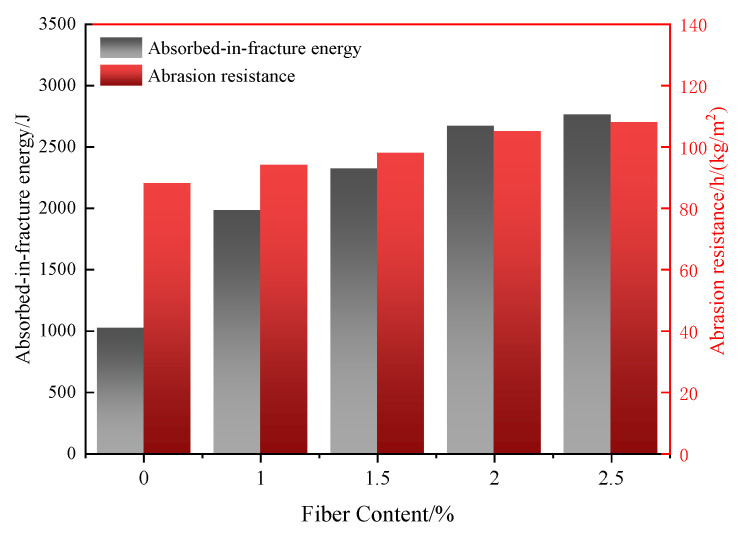
Absorbed-in-fracture energy and abrasion resistance of UHPC as a function of POM fiber content.

The impact and abrasion resistance of UHPC with different POM fiber contents are shown in Figure 6. It can be noticed that the impact and abrasion resistance of UHPC increases with the increase in POM fiber content. For the control sample, the absorbed-in-fracture energy and abrasion resistance of UHPC are 980 J and 88 h/(kg/m^2^), respectively. When the POM fiber content reaches 2.5%, the absorbed-in-fracture energy and abrasion resistance of UHPC are increased by 170.5% and 22.7%, which are 2760 J and 108 h/(kg/m^2^), respectively. The impact resistance is enhanced more than the abrasion resistance with POM fiber incorporation.

On the other hand, the improvement in POM fibers on the absorbed-in-fracture energy of UHPC diminishes after the POM content reaches 2%. Compared with the M2 sample, the impact resistance of the M2.5 sample only improves by 2.9%. The abrasion resistance is improved by only 2.6%. This implies that the increase in POM fiber shows a negligible reinforcing effect on UHPC after a content of 2%.

POM fiber in UHPC bridges cracks and restrains the matrix, which can enhance the material’s toughness to avoid stress concentration due to the blocking of shock waves [37]. POM fiber can also inhibit the emergence and expansion of cracks, reducing the wear and tear of the material, thus improving the impact resistance and abrasion resistance of UHPC. However, too much fiber addition would worsen its dispersion in UHPC. It is easy to for POM fibers to agglomerate and introduce defects, which weaken the impact and abrasion resistance of UHPC.

#### 3.1.4. Effect of POM Fiber Content on Flexural Toughness

**Figure 7:**
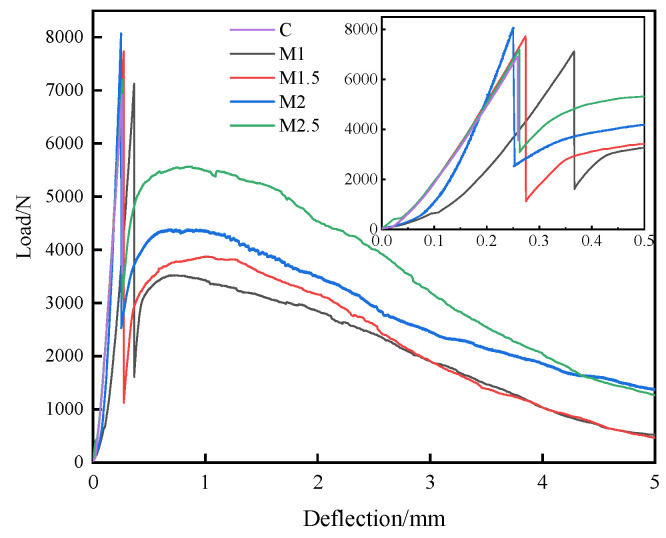
Load–deflection curves of UHPC with different POM fiber contents.

The load–deflection curves of UHPC with different POM fiber contents are given in Figure 7. It can be observed that the loading rapidly decreases after the peak value for the control sample, indicating the brittleness of the sample. The fracture behavior of UHPC with POM fibers exhibits a different fracture mode. Its loading–deflection curve also decreases abruptly after the peak, showing a secondary increase followed by a slow decrease. After that, the samples rupture. With increasing deflection, the UHPC continues to crack. After the peak loading, many cracks are already generated in the UHPC matrix. However, the POM fiber network in the UHPC slows its further damage. POM fiber can also consume external loading energy through fiber fracture and pull out, which increases the loading capacity of the specimen. Finally, the flexural toughness of the UHPC improves. It can be found that the peak loading of the UHPC gradually increases with an increasing amount of POM fiber from 1% to 2.5%. The secondary peak loading also becomes more prominent, and the area inside the curve increases gradually. This indicates that increasing POM fiber further enhances the flexural toughness of UHPC.

In order to quantitatively evaluate the effect of POM fiber on the toughness of UHPC, the toughness indices *I*_5_, *I*_10_, and *I*_20_ and the residual strength factors *R*_5,10_ and *R*_10,20_ of UHPC were calculated, with the results specified in Table 4. When the fiber content is 1%, *I*_5_, *I*_10_, and *I*_20_ are 3.2, 5.9, and 9.9, respectively, and *R*_5,10_ and *R*_10,20_ are 53.6 and 40.2, respectively. At a fiber content of 2.5%, *I*_5_, *I*_10_, and *I*_20_ improve by 37.5%, 50.8%, and 64.6%, respectively, and *R*_5,10_ and *R*_10,20_ improve by 67.3% and 84.8%, respectively. The enhancement in *I*_10_ and *I*_20_ is more remarkable than *I*_5_, which indicates that POM fiber has a more significant effect on improving the late toughness of UHPC specimens. When the fiber content increases from 2.0% to 2.5%, the *I*_5_, *I*_10_, and *I*_20_ of UHPC improve by 10%, 9.9%, and 11.6%, respectively. Overall, 2.0% of POM fiber obviously enhances the toughness of UHPC. The toughening effect of fiber on UHPC is still weak at a higher content than 2.0%.

### 3.2. Effect of Fiber Type

#### 3.2.1. Effect of Fiber Type on Workability

**Figure 8:**
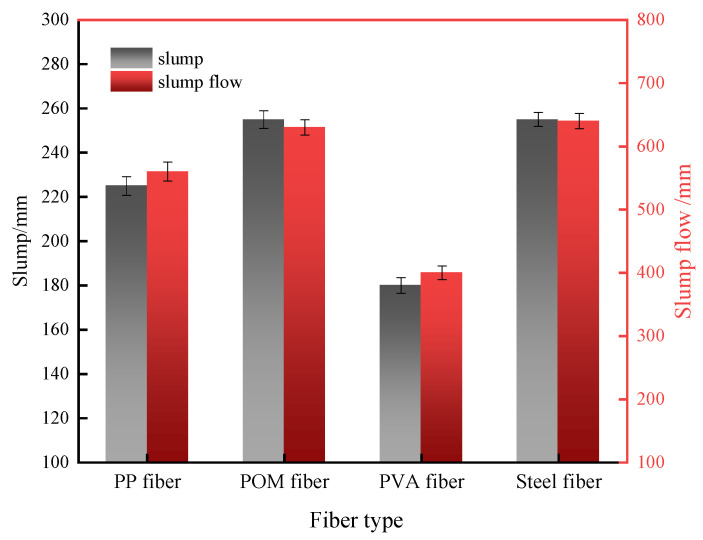
Workability of UHPC with different fibers.

The workability of UHPC prepared with different fibers is shown in Figure 8. It can be noticed that the UHPC with PVA fibers has the worst workability, with a slump flow of only 400 mm. The UHPC with steel fibers has the best workability. It has a slump and slump flow of 255 mm and 640 mm, respectively. The UHPC with POM fibers has good workability comparable to the steel fiber UHPC. The workability of UHPC with PP fiber has a slump and slump extension of 225 mm and 590 mm, respectively, which can still meet the requirements for self-compacting concrete [38]. PVA fiber has the most significant specific surface and the largest water requirement. Hence, its addition seriously worsens the workability of UHPC. PP fiber shows hydrophobicity and is vulnerable to agglomeration in the mixture, which also decreases the flowability of the concrete [39]. Steel fiber and POM fiber can uniformly disperse in UHPC, thus not negatively affecting the UHPC workability.

#### 3.2.2. Effect of Fiber Type on Mechanical Strength

**Figure 9:**
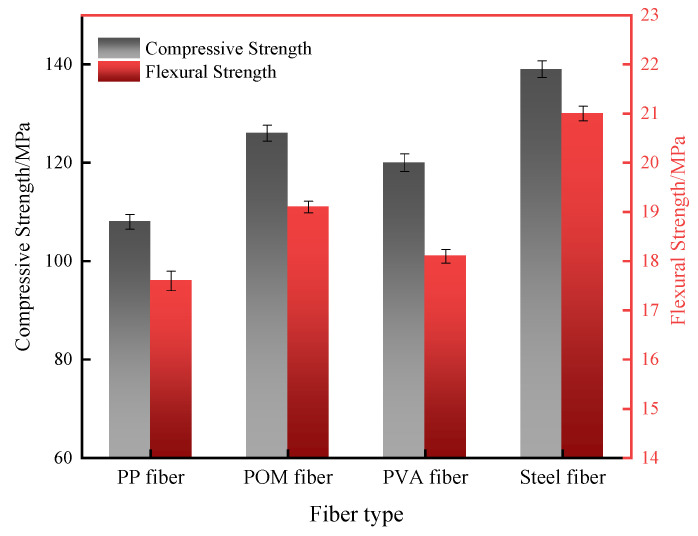
Mechanical strength of UHPC with different fibers at 28 d age.

**Figure 10:**
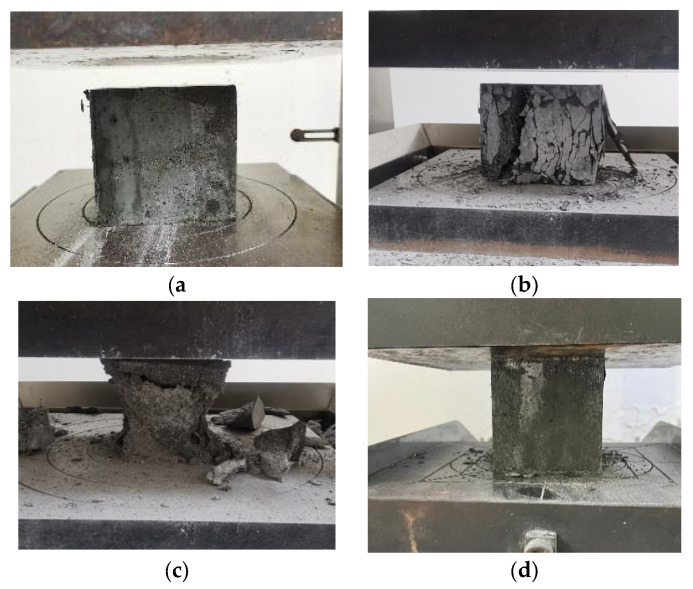
Effect of fibers on the failure mode of UHPC under compressive loading. (**a**) POM; (**b**) PP; (**c**) PVA; (**d**) SF.

The effect of fiber type on the 28 d mechanical properties of UHPC is shown in Figure 9. It can be seen that the UHPC with steel fibers had the highest 28 d compressive and flexural strengths, which reached 139 MPa and 21.0 MPa, respectively. The mechanical strength of the UHPC with synthetic fibers was distinctly lower than that of the UHPC with steel fibers. Nevertheless, UHPC with POM fibers shows the best mechanical strength among the synthetic-fiber-reinforced UHPC. It has 28 d compressive and flexural strengths of 126 MPa and 19.1 MPa, which are 6.8% and 8.5% higher than those of the PP fiber-reinforced UHPC and 3.3% and 5.5% higher than those of the PVA-fiber-reinforced UHPC, respectively. As expected, steel fibers have the highest elastic modulus and tensile strength, which can effectively constrain the deformation of UHPC and enhance its mechanical strength. Among the synthetic fibers, POM fibers have a higher modulus of elasticity and tensile strength than the other two fibers. Therefore, UHPC prepared with POM fibers shows the second highest mechanical strength. The damage mode of UHPC with different fiber types is illustrated in Figure 10. It should be noticed that UHPC with PVA fibers directly exploded under compressive loading, indicating the weak constraint of PVA fibers on the concrete. UHPC added with PP fibers ruptured with one penetrating crack and several obvious surface cracks. Conversely, UHPC reinforced with steel and POM fibers did not have significant splitting cracks or splattering debris upon damage, demonstrating their excellent ability to toughen the concrete.

#### 3.2.3. Effect of Fiber Type on Impact and Abrasion Resistance

**Figure 11:**
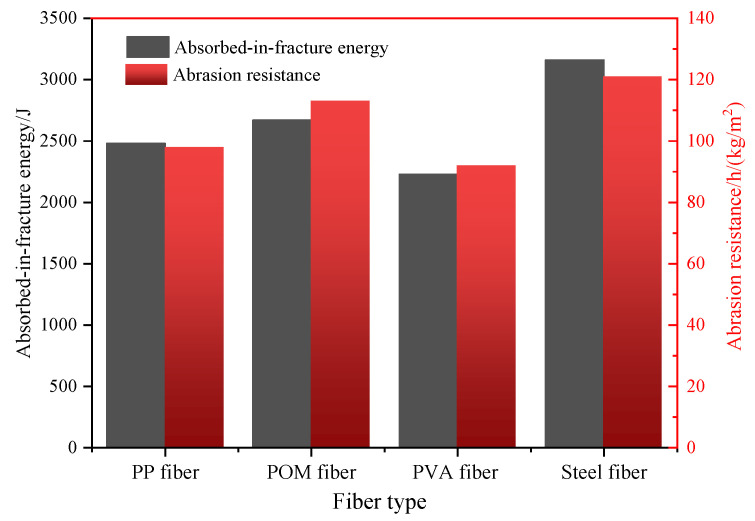
The absorb-in-fracture energy and abrasion resistance of UHPC with different fibers.

The effect of fiber type on the impact and abrasion resistance of UHPC is shown in Figure 11. It can be observed that UHPC prepared from steel fibers has the highest absorbed-in-fracture energy and abrasion resistance. The values are 105 h/(kg/m^2^) and 3160 J. Steel fiber has high stiffness and an interfacial solid bond with the cement matrix, which produces an obvious bond connection with cement paste. Therefore, steel fiber can absorb the largest energy under impact and abrasion. Compared with the UHPC with steel fibers, the absorbed-in-fracture energy of UHPC with POM, PP, and PVA fibers was reduced by 7.1%, 15.5%, and 14.2%, and the abrasion resistance was reduced by 21.5%, 15.9%, and 29.4%, respectively. POM fibers had the highest absorbed-in-fracture energy and abrasion strength among the synthetic fibers. PP fibers were the second highest. This is due to the low interfacial bond strength between PP fibers and cement matrix. PVA-fiber-strengthened UHPC shows the lowest impact and abrasion resistance, which may be derived from the low tensile strength of PVA fibers. POM fiber has a high elastic modulus, which can effectively bridge the cracks and prevent them from growing and propagating. POM fiber can also adsorb impact and abrasion energy through pull out and breakage.

#### 3.2.4. Effect of Fiber Type on Flexural Toughness

**Figure 12:**
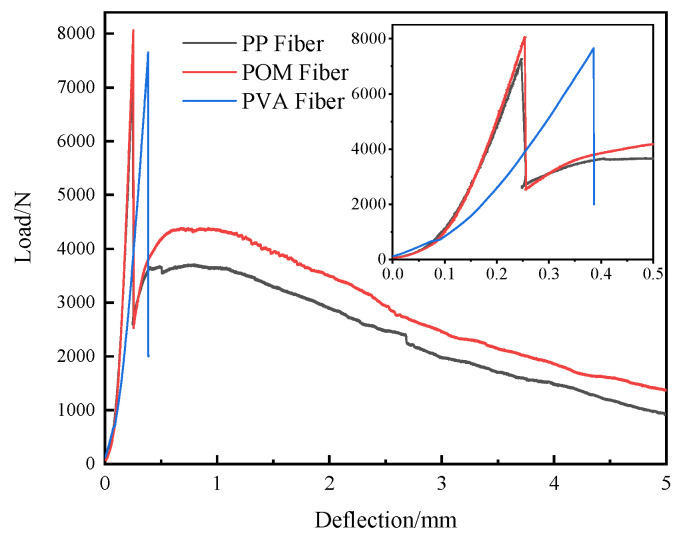
Load–deflection curves of UHPC with different POM fiber types.

Steel-fiber-reinforced UHPC shows a higher flexural strength than synthetic fiber-reinforced UHPC, so flexural toughness studies were only performed on synthetic fibers. Figure 12 shows the load–deflection curves of UHPC with different synthetic fibers. It can be noticed that the load–deflection curve of the PVA specimen is directly interrupted after the sudden drop, indicating the breakage of the UHPC. Conversely, both the curves of PP and POM2 specimens show plateaus after the peak loading. This demonstrates that both PP and POM fibers can improve the flexural toughness of UHPC. Compared with the UHPC prepared by PP fibers, the load–deflection curve of UHPC with POM fibers has a higher peak loading and higher loading at the plateau areas.

Moreover, the plateau area of the UHPC specimen with POM fibers shows more jags, which is caused by the pull out and breakage of POM fibers [40]. The sound of pulling and breaking POM fibers can be clearly heard during the test.

The toughness indices and residual strength factors of UHPC with different fibers were calculated, with the values listed in Table 5. As shown in the table, PVA-fiber-reinforced UHPC has the most minor toughness indices of *I*_5_, *I*_10_, and *I*_20_. Due to the entire breakage of the sample, UHPC with PVA fiber shows zero residual strength. UHPC with POM fiber has the highest toughness indices and residual strength, indicating POM fiber’s most pronounced toughening effect among the synthetic fibers.

### 3.3. Microstructure of Fiber-Reinforced UHPC

**Figure 13:**
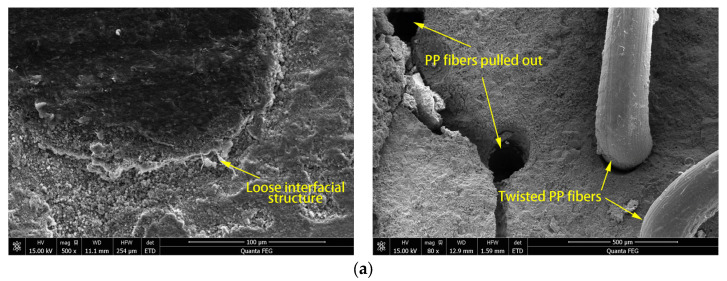

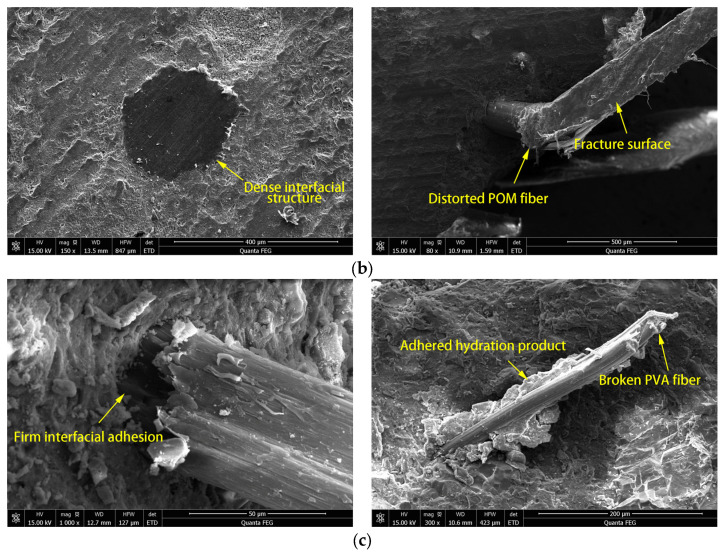
SEM images of interfaces between fibers and UHPC paste. (**a**) PP fiber; (**b**) POM fiber; (**c**) PVA fiber.

Figure 13 shows SEM images of the interface between different synthetic fibers and UHPC paste. It can be observed that the UHPC paste around the PP fibers is loose, and there are clusters of hydration products. The PP fiber at the fracture surface was twisted, but its structure was intact, and its surface was smooth. In addition, round holes on the fracture surface were presented, caused by the pulling out of the whole fiber. This indicates the weak bonding between PP fibers and UHPC paste. PP fiber reinforcement can only absorb the tensile loading through friction after debonding from the paste [41]. This part of energy absorption is finite, which leads to the weak bridging effect of PP fibers in the UHPC. The UHPC paste around POM and PVA fibers is dense, and there is no apparent interfacial transition zone, especially the PVA fiber attached to the hydration products, indicating that these fibers are well bonded with the UHPC paste. POM fiber in the fractured surface is distorted and torn apart along its axial direction. The fracture surface is more extensive along the axial direction than the direction perpendicular to the axis, indicating that POM fiber is firmly bonded with the paste. When UHPC is damaged, reinforced POM fibers can absorb energy through debonding, fracture, and tearing, which effectively bridge the cracks in the UHPC paste. The bond between the PVA fiber and the paste is also firm. However, due to the low tensile strength of PVA fiber, most of the fibers are simultaneously broken upon UHPC fracture. Therefore, the toughening effect of PVA fiber on UHPC is not obvious.

### 3.4. Comparison between Different Fibers

Among these fibers, steel fibers have the highest elastic modulus and tensile strength, which can effectively constrain the deformation of UHPC and enhance its mechanical strength. Therefore, UHPC prepared with steel fibers undoubtedly had the best mechanical, impact, and abrasion properties.

The mechanical features of UHPC with synthetic fibers were distinct from UHPC with steel fibers. PVA fiber has the highest specific surface and the largest water requirement [42]. Hence, its addition seriously worsens the workability of UHPC. Although PVA fiber has good interfacial connection with the UHPC paste, its low tensile strength limited the toughness of UHPC. Therefore, PVA-fiber-strengthened UHPC shows the lowest impact and abrasion resistance. PP fiber shows hydrophobicity and is vulnerable to agglomeration in the mixture, which also decreases the flowability of UHPC. Additionally, PP fiber shows the weakest binding to UHPC paste among synthetic fibers, which can be directly pulled out of the matrix during cracking. This limits the toughening effect of PP fiber. POM fiber has a higher modulus of elasticity and tensile strength than the other two fibers and it also shows good bonding with the cement matrix. Hence, POM fiber can effectively bridge the cracks and prevent them from growing and propagating. POM fiber can also adsorb impact and abrasion energy through pull out and breakage. UHPC with POM fibers had the most satisfying workability, mechanical, impact, and abrasion properties among the synthetic fibers.

## 4. Conclusions

This work investigates the influence of the mechanism of POM fiber on the workability, mechanical properties, impact, and abrasion resistance of UHPC. The toughening effect of POM fiber on UHPC is compared with other fibers. Conclusions can be made as follows:

1. With the increase in POM fiber content, the slump and slump flow of UHPC decrease gradually. The compressive and flexural tensile properties of UHPC first increase and then decrease as POM fiber content increases. Its mechanical strength reaches the highest at 2% POM fiber. Increasing POM fibers can improve the flexural toughness, impact, and abrasion resistance of UHPC.
2. Among four types of fibers, UHPC prepared with steel fibers had the best mechanical, impact, and abrasion properties. UHPC with POM fibers had the most satisfying workability, mechanical, impact, and abrasion properties among the synthetic fibers.
3. Microstructure investigation found that PP fiber has the weakest binding with UHPC paste. The hardened paste around the PP fiber shows a loose structure and high porosity. When UHPC is cracked, PP fiber is directly pulled out of the matrix. POM and PVA fibers have a better interfacial connection with the UHPC paste, which can effectively bridge the cracks. However, the low tensile strength of PVA fiber limits the toughness of UHPC. POM fiber has a high tensile strength and can absorb tensile loading through debonding, fracture, and tearing, indicating its significant role in toughening the UHPC.

## Figures and Tables

**Table 1:** Chemical composition of cementitious materials.

Type	SiO_2_	Al_2_O_3_	Fe_2_O_3_	CaO	MgO	SO_3_	K_2_O	Na_2_O	TiO_2_
Cement	21.93	4.45	5.97	61.90	1.35	2.24	0.52	0.16	0.26
Silica Fume	93.05	0.48	0.13	0.79	0.33	0.75	0.14	0.07	0
Fly ash	62.6	19.71	3.72	6.28	0.97	0.13	1.36	1.22	0.67

**Table 2:** Specifications of fibers.

Specification	POM Fiber	PP Fiber	PVA Fiber	Steel Fiber
Morphology	monofil	monofil	cluster	monofil
Apparent density/kg·m^−3^	1420	920	1270	7850
Modulus/Gpa	>8	>3.5	-	200~220
Length/mm	13	12	12	13
Diameter/mm	0.2	0.2	2dtex	0.2
Strength/Mpa	≥967	≥600	cn/dtex ≥ 12	≥2200
Elongation rate/%	≥18	≥15	6–8	≥10

**Table 3:** Mixing proportion of UHPC.

No.	Cement/kg/m^3^	Silica Fume/kg/m^3^	Fly Ash/kg/m^3^	Quartz Sand/kg/m^3^	Water/kg/m^3^	Superplasticizer/kg/m^3^	Fiber Type	Fiber Volume/%
C	800	200	150	1000	184	28.75	—	0
POM1							POM	1.0
POM1.5							POM	1.5
POM2							POM	2.0
POM2.5							POM	2.5
PP							PP	2.0
PVA							PVA	2.0
SF							SF	2.0

**Table 4:** The toughness indices and the residual strength factors of UHPC with different amount of POM fiber.

No.	*I*_5_	*I*_10_	*I*_20_	*R*_5,10_	*R*_10,20_
C	1	1	1	0	0
M1	3.2	5.9	9.9	53.6	40.2
M1.5	3.1	6.1	10.7	60.7	45.4
M2	4.0	8.1	14.6	81.1	65.6
M2.5	4.4	8.9	16.3	89.7	74.3

**Table 5:** The toughness indices and the residual strength factors of UHPC with different types of fibers.

Type	*I*_5_	*I*_10_	*I*_20_	*R*_5,10_	*R*_10,20_
PP fiber	3.9	7.6	13.6	74.3	60.1
POM fiber	4.0	8.1	14.6	81.1	65.6
PVA fiber	1	1	1	0	0

## Data Availability

The data presented in this study are available in article.
